# Facile Formation of Multifunctional Biomimetic Hydrogel Fibers for Sensing Applications

**DOI:** 10.3390/gels10090590

**Published:** 2024-09-13

**Authors:** Mengwei Jia, Mingle Guan, Ryan Yao, Yuan Qing, Xiaoya Hou, Jie Zhang

**Affiliations:** 1 School of Mechanical Engineering, Jiangnan University, Wuxi 214122, China; 2 Jiangsu Key Laboratory of Advanced Food Manufacturing Equipment and Technology, Jiangnan University, Wuxi 214126, China; 3 College of Engineering, University of Illinois at Urbana-Champaign, Champaign, IL 61820, USA

**Keywords:** microfluid, coaxial co-extrusion, complex structure, multiple functions, biomimetic hydrogel fibers

## Abstract

To face the challenges in preparing hydrogel fibers with complex structures and functions, this study utilized a microfluidic coaxial co-extrusion technique to successfully form functional hydrogel fibers through rapid ionic crosslinking. Functional hydrogel fibers with complex structures, including linear fibers, core–shell structure fibers, embedded helical channels, hollow tubes, and necklaces, were generated by adjusting the composition of internal and external phases. The characteristic parameters of the hydrogel fibers (inner and outer diameter, helix generation position, pitch, etc.) were achieved by adjusting the flow rate of the internal and external phases. As biocompatible materials, hydrogel fibers were endowed with electrical conductivity, temperature sensitivity, mechanical enhancement, and freeze resistance, allowing for their use as temperature sensors for human respiratory monitoring and other biomimetic application developments. The hydrogel fibers had a conductivity of up to 22.71 S/m, a response time to respiration of 37 ms, a recovery time of 1.956 s, and could improve the strength of respiration; the tensile strength at break up to 8.081 MPa, elongation at break up to 159%, and temperature coefficient of resistance (TCR) up to −13.080% °C^−1^ were better than the existing related research.

## 1. Introduction

Hydrogels are formed from crosslinking monomers or polymers to form 3D networks that can retain large amounts of water. Usually, hydrogels are sparsely crosslinked, making them soft and flexible like the soft tissues of living organisms [1,2,3]. However, the structures and properties of hydrogels can be modulated by adjusting synthetic materials, crosslinking methods, and functional additives. This versatility makes them highly adaptable to a large number of applications such as motion monitoring, soft robotics, personalized medicine, and optical detection [4,5,6,7,8,9,10,11,12,13,14,15,16,17,18,19,20,21,22,23,24,25,26].

Hydrogel fibers, as a biocompatible fibrous material, have attracted attention for their wide range of applications in the field of tissue engineering [27]. Soft and stretchable conductive hydrogel fibers can be distributed in flexible electronic devices like the vascular network in human tissues, playing important roles in transmitting information [28]. On this basis, conductive hydrogel fibers also need to have good mechanical strength, freeze resistance, and moisturizing properties to meet flexibility and environmental stability requirements for flexible sensing applications [29,30,31,32]. Studies have shown that the partial replacement of water in hydrogels with organic solvents or ionic liquids could confer good freeze resistance and moisturizing properties [33,34,35,36,37,38,39]. Conductive hydrogel fibers have also been endowed with environmental responsiveness for artificial intelligence in human–machine interfaces [40,41,42,43].

Furthermore, hollow hydrogel fibers can be permeable storage devices for controllable delivery systems, soft body driving systems, and biological scaffolds [44]. In recent years, they have been used for controlled drug delivery, cell culture, substance transport, in vitro organ cultivation, and in vivo cell environment simulation [45,46,47,48,49,50]. Hollow hydrogel fibers have been used to mimic the complex cellular microenvironment in organisms and to control cell culture [51]. Inspired by blood vessels and intestines, researchers prepared microscopic hollow hydrogel fibers and demonstrated their potential applications in vascular transplantation [52]. They also show promise with complex structures and specific shapes as they are flexible and biomimetic materials in smart wearables [45].

It is undeniable that some relevant studies have indeed been reported to prepare hydrogel fibers with complex structures. However, these studies required expensive and complicated experimental equipment, such as microfluidic chips, which have the common problems of easy blockage, low efficiency, and high cost. Moreover, the preparation process of hydrogel fibers with different structures was highly dependent on the equipment, and often, a set of equipment could only prepare hydrogel fibers with one specific structure. However, low cost and easy operation are the keys for a new technology to move from theory to practical application and to achieve large-scale dissemination, especially in less developed regions where the new technology is needed more.

Microfluidic techniques are commonly used for the preparation of hydrogel fibers. The microfluidic system, which can be continuously extruded, is a simple and flexible preparation process. It allows for controlled sizes and shapes and has a low cost compared with template methods, direct laser writing, wet spinning, dry spinning, and electrostatic spinning. Currently, conventional microfluidic devices are effective in preparing axially homogeneous hydrogel fibers but struggle to prepare hydrogel fibers with complex channel structures on micro- and nanoscales.

The principles for the selection of the hydrogel fiber material system in this study were, firstly, the biocompatibility, non-toxicity, non-harmfulness, greenness, and controllable cost of the material; secondly, the processing and molding performance of the material, which could be simply and quickly crosslinked and molded under mild and controllable experimental conditions; and lastly, the functionality, which conferred one or more functionalities to the hydrogel fibers by the integration of the material’s own properties. Among many choices of hydrogels, thermo-responsive poly (N-isopropylacrylamide) (PNIPAM) has attracted great attention due to its significant thermal behavior around a phase transition temperature (PTT) of 32 °C in water, which is near human body temperature [53]. Around the PTT, the hydrogel can achieve a rapid yet reversible aqueous phase transition; therefore, it can be used to develop biomedical and bioengineering applications such as biomarkers, injectable stents, and drug carry/delivery agents. Commercially available Poly (3,4-ethylenedioxythiophene): poly (4-styrenesulfonate) (PEDOT:PSS) is water soluble, highly conductive and stabile, light translucent, and biocompatible and is an ideal additive for water-based hydrogel systems [53]. Polyvinyl alcohol (PVA) is a polymer material with good water solubility and biocompatibility, which can be formed into physically crosslinked hydrogels by a simple freeze–thaw treatment. NaCl can produce a salting-out effect, which induces the intertwining of PVA molecular chains and the formation of a more compact molecular network structure, thus improving the mechanical properties of the hydrogel, as well as increasing its electrical conductivity [42]. In the water/glycerin binary solvent mixture, a large number of hydrogen bonds are formed between the water molecules and the hydroxyl groups in the glycerin, which can lock the water molecules firmly in place, resulting in a low freezing point and evaporation rate, and therefore, good frost resistance and moisturizing properties [1,4].

In order to promote the rapid development of multifunctional biomimetic hydrogel fibers, a new microfluidic coaxial co-extrusion scheme was proposed in this study. The base mechanism was similar to the rapid ionic crosslinking of calcium alginate hydrogels. Further adjusting the composition and flow rate of the internal and external phase solutions allowed for the rapid and controllable preparation of biomimetic hydrogel fibers with complex structures, including linear fibers, core–shell structure fibers, embedded helical channels, hollow tubes, and necklaces. The fiber’s characteristic parameters (inner and outer diameter, helix generation position, pitch, etc.) were adjusted by the flow rates of the internal and external phases.

The main component of the hydrogel fibers was sodium alginate. Its biocompatibility allowed for their uses in temperature sensing and human respiratory monitoring and provided a reference for further biomimetic applications. Various additives conferred different properties: Poly (3,4-ethylenedioxythiophene): poly (4-styrenesulfonate) (PEDOT:PSS) increased electrical conductivity; Poly (N-isopropylacrylamide) (PNIPAM) added thermal responsiveness; Polyvinyl alcohol (PVA), NaCl, and sodium alginate (Na-Alg) enhanced the mechanical properties; and a water/glycerol binary solvent provided freeze resistance. Combining an instantaneous ionic crosslinking reaction and the automatic winding device, the developed scheme could realize large-scale and automatic continuous production of multifunctional biomimetic hydrogel fibers.

## 2. Results and Discussion

### 2.1. Design and Preparation of Multifunctional Biomimetic Hydrogel Fibers

#### 2.1.1. Design and Preparation of Linear Hydrogel Fibers

**Figure 1:**
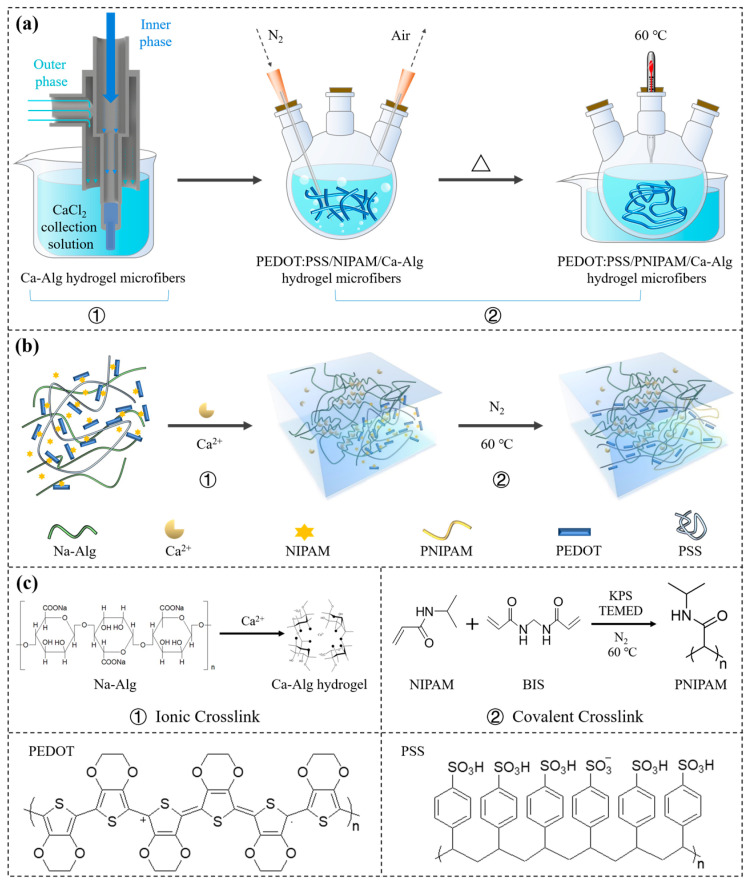
(**a**) Schematic of the preparation of linear hydrogel fibers with temperature-sensitive conductive function. (**b**) Schematic diagram of hydrogel fibers prepared by ionic crosslinking and covalent crosslinking. (**c**) Reaction equation of ionic crosslinking and covalent crosslinking.

A schematic of the microfluidics to generated the hydrogel fibers was shown in Figure 1(a①). The inner-phase solution consisted of 2.5 mL of diH_2_O, 2.5 mL of aqueous PEDOT:PSS (1.5 wt%), 5.0 g of aqueous sodium alginate (2 wt%), 0.01 g of crosslinker BIS, 0.01 g of initiator KPS, 0.05 mL of accelerator TEMED, and 0.5 g of NIPAM temperature-sensitive monomers. The external phase solution and the collection solution were both 5 wt% aqueous calcium chloride. The flow rates of both the inner and outer phases were 120 µL/min.

Hydrogel fibers were prepared in two steps. In the first step, the inner-phase solution and outer-phase solution were coaxially co-extruded into the collection solution by a microfluidic device (Figure 1a①). Sodium alginate in the inner-phase solution and Ca^2+^ in the outer-phase solution rapidly ionically crosslinked into a Ca-Alg hydrogel (Figure 1(c①)), which resulted in the PEDOT:PSS/NIPAM/Ca-Alg hydrogel microfibers (Figure 1(b①)).

In the second step, the collection solution and the hydrogel microfibers were transferred together into a sample bottle, which was purged with N_2_ for deoxidation for 15 min. The bottle was then heated in a 60 °C water bath for about 5 min while kept air-tight to maintain an oxygen-free environment (Figure 1(a②)). The KPS initiator rapidly decomposed to produce a large number of free radicals, which reacted with the NIPAM monomer dispersed in the Ca-Alg network through free radical polymerization to form linear PNIPAM polymer chains. A covalently crosslinked network was formed under the action of a BIS crosslinking agent (Figure 1(c②)). The covalently crosslinked network of PNIPAM and the Ca-Alg network formed an IPN of PEDOT:PSS/PNIPAM/Ca-Alg hydrogel microfibers (Figure 1(b②)).

#### 2.1.2. Design and Preparation of Core–Shell and Anti-Freeze Hydrogel Fibers

The anti-freeze hydrogel precursor solution was 4 g of PVA, 12 mL of ultrapure water, and 28 g of glycerol, which was heated at 85 °C and stirred at 80 rpm for 4 h and then left to cool down to obtain a homogeneous and transparent inner-phase solution. In the water/glycerol binary mixture, hydrogen bonds were formed between water molecules and hydroxyl groups in glycerol [1]. A large number of hydrogen bonds also existed between PVA and hydroxyl groups in glycerol [25,26]. The large number of hydrogen bonds lock the water molecules firmly, resulting in a lower freezing point and evaporation rate and, therefore, good anti-freezing and moisturizing properties [1,4]. 

**Figure 2:**
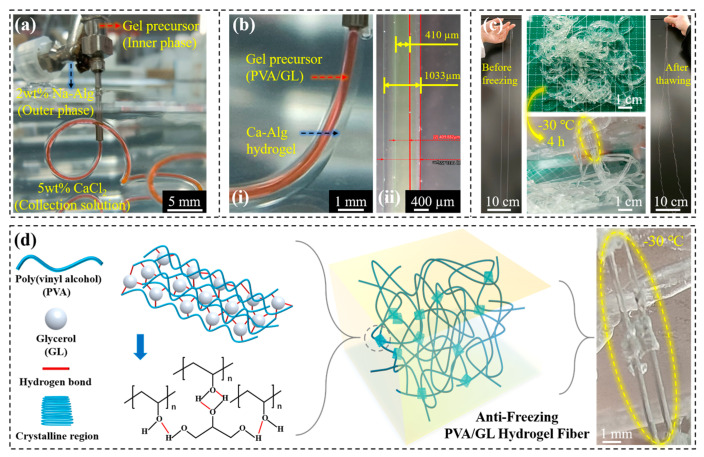
(**a**,**b**) Preparation of core–shell structured hydrogel fibers, in which the core was red stained for easy observation. (**c**) Preparation of anti-freeze hydrogel fibers. (**d**) The molding mechanism of anti-freeze hydrogel fibers, freezing promoted the formation of microcrystalline regions of PVA and the formation of a large number of hydrogen bonds between the hydroxyl groups of PVA and propanetriol, so that the hydrogel fibers had good mechanical and anti-freeze properties.

As shown in Figure 2, the process of preparing anti-freeze hydrogel fibers by using core–shell hydrogel fibers was mainly divided into three steps.

Firstly, as shown in Figure 2a, the pre-configured inner-phase solution (anti-freeze hydrogel precursor solution) and outer-phase solution (2 wt% aqueous Na-Alg) were co-extruded into the solidification collection solution (5 wt% aqueous CaCl_2_) through coaxial needles at a certain flow rate ratio, which was controlled by a high-precision digital syringe pump (Figure S1, Supporting Information).

Then, as shown in Figure 2b, at the moment when the inner- and outer-phase solutions were co-extruded into the solidification collection solution, the ionic crosslinking of Na-Alg and Ca^2+^ formed a Ca-Alg hydrogel, which acted as a solidified outer shell structure and wrapped around the inner-phase solution to form a core–shell hydrogel fiber, with Ca-Alg surrounding the anti-freeze precursor solution (Movie S1, Supporting Information), which could be rolled up and collected by the winding device (Movie S2, Supporting Information).

Finally, as shown in Figure 2c, the core–shell hydrogel fibers were subjected to a freeze–thaw treatment. As shown in Figure 2d, the large number of hydrogen bonds between PVA and glycerol and the microcrystalline region of PVA both acted to form a physically crosslinked hydrogel fiber. The core gel fibers remained flexible after freezing, while the Ca-Alg shell became brittle after freezing and was easily stripped off (Figure S2, Supporting Information).

#### 2.1.3. Design and Preparation of Hydrogel Fibers with Embedded Helical Channels

**Figure 3:**
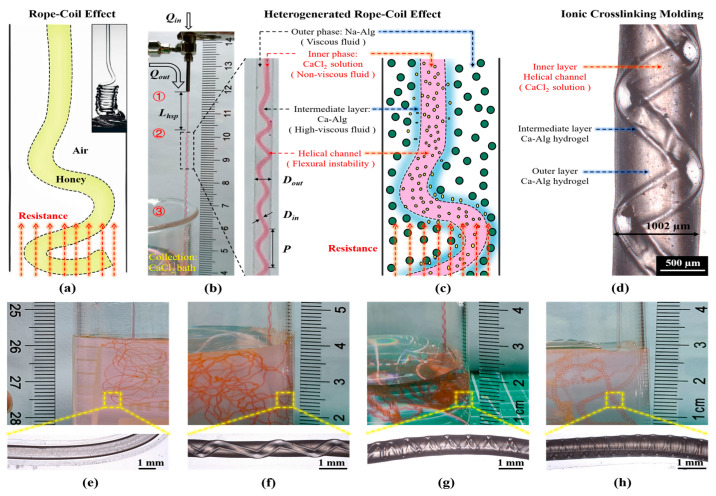
Fabrication of hydrogel fibers with embedded helical channel based on heterogenerated rope coil effect [52]: (**a**) Schematic diagram of rope coil effect. (**b**) Continuous preparation of calcium alginate hydrogel fibers containing helical channels using a homemade coaxial co-extruder (*Q_in_* is the internal phase solution flow rate, *Q_out_* is the external phase solution flow rate, *D_hsp_* is the helix-starting position, *D_out_* is the diameter of the calcium alginate hydrogel fiber, *D_in_* is the diameter of the embedded helical channel, *p* is the pitch of the helical channel, and the internal phase calcium chloride aqueous solution is dyed red). (**c**) Schematic diagram of heterogenerated rope coil effect. (**d**) Optical magnification of the embedded helical channel hydrogel fibers after CaCl_2_ coagulation collection bath. (**e**–**h**) Physical and optical magnification of hydrogel fibers with different channels embedded.

As shown in Figure 3a, the coiling rope effect refers to the viscous liquid flowing down from an appropriate height to form a small liquid column, which bends and becomes unstable due to axial resistance after contact with the plane and spirals itself into a coil type, which can be used to prepare spring shaped hydrogel fibers [46].

As shown in Figure 3b–c, Ca-Alg hydrogel fibers with embedded helical channels were prepared based on the heterogenerated coiled rope effect (Movies S3 and S4, Supporting Information) [52]. Moreover, 50 mM aqueous CaCl_2_ as the inner phase, 2 wt% aqueous Na-Alg as the outer phase, and 100 mM aqueous CaCl_2_ as the coagulation collection bath were used (Figure S3, Supporting Information).

In the first stage, the viscous fluid in the outer phase was coaxially co-extruded with the non-viscous fluid in the inner phase to create laminar flow. As shown in Figure 3(b①), viscous Na-Alg as the outer phase and non-viscous CaCl_2_ as the inner phase were coaxially co-extruded with a certain flow rate ratio (*Q_out_/Q_in_*). The non-viscous fluid flowed inside the viscous fluid to form a stable laminar flow, at which time a coaxial linear structure with Na-Alg as the shell and CaCl_2_ as the core was produced.

In the second stage, an ionic crosslinking reaction occurred at the interface of the inner and outer phases to generate an intermediate layer (highly viscous fluid). This underwent flexural destabilization when its flow was subjected to a certain resistance, leading to a helical structure. As shown in Figure 3(b②), during the coaxial co-extrusion process, the Ca^2+^ of the inner phase continued to diffuse into the Na-Alg of the outer phase, and the ionic crosslinking reaction continued at the laminar flow interface between the inner- and outer-phase solutions, rapidly forming the highly viscous Ca-Alg intermediate layer.

At this point, the principles of the heterogenerated coiling rope effect (Figure 3c) were similar to those of the coiling rope effect (Figure 3a) in classical fluid dynamics [46]. In the coiling rope effect, a higher viscosity fluid flows within a lower viscosity fluid. When subjected to a sufficiently large axial resistance, flexural instability leads the inner fluid to form a helical structure (Figure 3a). In this experiment, it was the Ca-Alg (highly viscous fluid) in the middle layer surrounded by less viscous Na-Alg that underwent flexural instability, which in turn produced the helical structure.

In the third stage, as shown in Figure 3(b③), upon entering the CaCl_2_ coagulation collection bath, the Na-Alg in the outer phase underwent further ionic crosslinking reactions with Ca^2+^ to form a Ca-Alg hydrogel shell (Figure 3d). The aqueous CaCl_2_ in the inner phase continued flowing, allowing the generated helical channels to remain hollow, which resulted in the formation of Ca-Alg hydrogel fibers with embedded helical channels (Figure 3c,d). In addition, it was found during the experiments that using different flow rate ratios (*Q_out_/Q_in_*) changed the tightness of the generated helices (Figure 3e–h). 

#### 2.1.4. Design and Preparation of Hollow Tubular Hydrogel Fibers

**Figure 4:**
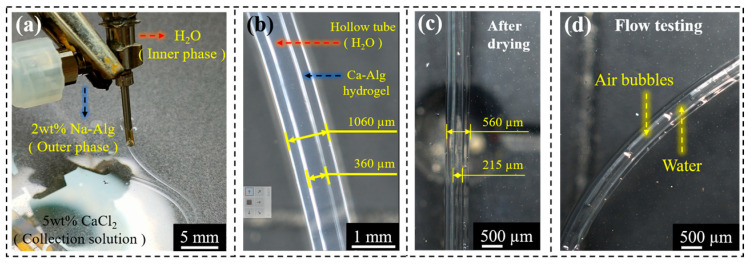
Preparation of hollow tubular hydrogel fibers (**a**), characteristic dimensions (**b**,**c**), and demonstration of circulation (**d**).

As shown in Figure 4a,b, hollow tubular hydrogel fibers could be produced by using a coaxial co-extrusion device with water as the inner phase, Na-Alg solution (2 wt%) as the outer phase, and CaCl_2_ solution (5 wt%) as the coagulation collection bath. As shown in Figure 4c, after drying, Ca-Alg hydrogel fiber lost water and became thinner but could still maintain the hollow tubular structure. Further, it was tested for liquidity by injecting water (Figure 4d). In addition, according to the above experimental results, it could be inferred that the inner and outer diameter of the hollow tubular hydrogel fiber could also be regulated by the internal and external phase flow rate.

#### 2.1.5. Design and Preparation of Necklace-Shaped Hydrogel Fibers

**Figure 5:**
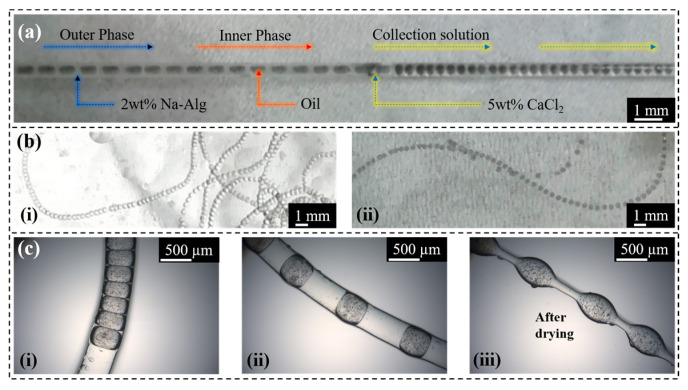
(**a**) Preparation of necklace-shaped hydrogel fibers, with black staining of the oil phase for observation. (**b**) (**i**,**ii**) Physical photographs of necklace-shaped hydrogel fibers. (**c**) (**i**–**iii**) (**i**,**ii**) Modulation of the spacing of embedded oil-phase microspheres by adjusting the flow rate of the internal-phase solution; (**iii**) Change in the shape of hydrogel fibers after drying.

As shown in Figure 5a,b, the preparation of hydrogel fibers with embedded helical channels was simplified to produce necklace-shaped hydrogel fibers by using oil as the inner phase, Na-Alg solution (2 wt%) as the outer phase, and CaCl_2_ solution (5 wt%) as the coagulation collection bath. As shown in Figure 5c(i,ii), the spacing of the embedded oil phase microspheres could be regulated by adjusting the internal phase solution flow rate. As shown in Figure 5c(iii), the shape of the necklace-shaped hydrogel fibers could be further changed after drying.

### 2.2. Characterization of Multifunctional Biomimetic Hydrogel Fibers

#### 2.2.1. Characterization of Linear Hydrogel Fibers

**Figure 6:**
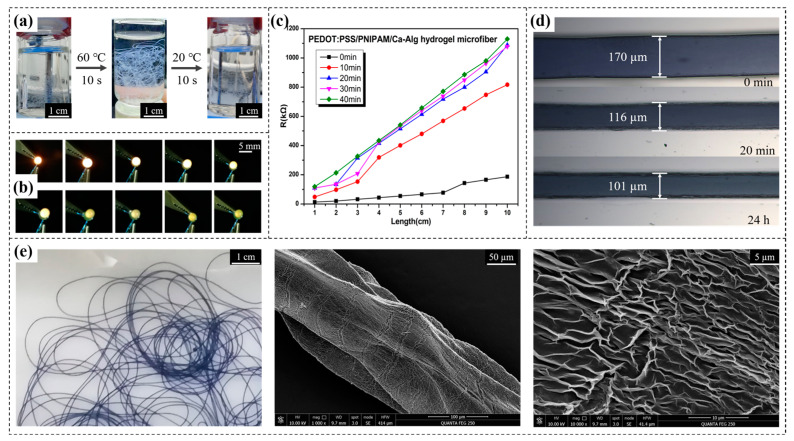
Testing characterization of hydrogel microfiber: (**a**) Temperature response of PNIPAM/Ca-Alg hydrogel microfiber demonstrated. (**b**) Conductivity demonstration of PEDOT:PSS/PNIPAM/Ca-Alg hydrogel microfiber. (**c**) Variation of sample resistance with time and length. (**d**) Hydrogel microfiber lost water and became thin. (**e**) Physical demonstration and microscopic morphology of hydrogel microfiber.

Temperature Sensitivity Test. As shown in Figure 6a, in order to facilitate the observation of state change during the temperature response test for the hydrogel fibers, PNIPAM/Ca-Alg hydrogel microfibers without PEDOT:PSS were prepared (Figure S4, Supporting Information). When heated for 10 s at 60 °C in a water bath, the fibers changed from translucent to milky white, and they quickly became translucent again after cooling for 10 s at room temperature (20 °C). The observed transition demonstrated that the fabricated hydrogel fibers could achieve a rapid and reversible temperature response, a sign of successful IPN formation. 

Electrical Conductivity Test. As shown in Figure 6b, PEDOT:PSS/PNIPAM/Ca-Alg hydrogel microfiber was connected in series to a 9 V circuit, and the change in the brightness of the yellow light-emitting diode was observed when connected at different lengths. It was judged that the hydrogel fiber was conductive and that its resistance increased with an increase in length.

Further, an LCR circuit was used to test the resistance of the hydrogel fibers. For all test conditions, 1 V and 1 kHZ were used. The measured resistance could be used to determine the conductivity σ (S/m) of the fibers using the equation *σ* = *L*(*RS*)^−1^, where *R* is the measured resistance and *L* and *S* are the length and cross-sectional area of the fiber, respectively. As shown in Figure 6c, the resistance was positively correlated with both length and time, with the relationship between resistance and length also being linear.

As shown in Figure 6d, the hydrogel fibers would lose water and become thin when left out, and the electrical conductivity of the hydrogel fibers would be redetermined at different times. One of the reasons for the increase in resistance over time was the decrease in fiber diameter due to water loss, which occurred mainly in the first 20 min. After this, the resistance barely varies with time, i.e., the water content of the sample equilibrated with the surrounding vapor (18 °C, 66% RH). The diameter of the hydrogel fibers decreased from 170 µm to 110 µm. Another reason for the increase in electrical resistance over time was the decrease in conductivity due to water loss. The conductivity of the PEDOT:PSS/PNIPAM/Ca-Alg hydrogel microfibers when hydrated and equilibrated were 22.71 S/m and 9.05 S/m, respectively, showing a 60.1% decrease in conductivity after water was lost.

The vast majority of hydrogels are ion-conducting, and their conductivity is generally below 1 S/m, severely limiting their application in bioelectronics. It is difficult for the existing conductive hydrogels to simultaneously meet the electrical, mechanical, biocompatibility, and stability requirements of practical applications. The development of high-performance, high-efficiency, and long-term stable hydrogels is one of the most important challenges in the field of human–machine interface interaction and integration. Prof. Zhao Xuanhe reported a simple and effective method to prepare high-performance conductive polymer hydrogels and successfully prepared pure PEDOT:PSS hydrogels with conductivity as high as 40 S/cm, one of the highest values of hydrogel conductivity at present, and pointed out that the formation of stable conductive polymer network was the key to obtain high-performance conductive polymer hydrogels.

As shown in Figure 6e, the surface morphology of the PEDOT:PSS/PNIPAM/Ca-Alg hydrogel microfibers was observed by SEM after sample preparation with freeze-drying and application of a gold coating. The fibers consisted of dense and uniformly distributed 3D networks (interpenetrating polymer network, IPN) and showed a certain orientation along the fiber length, likely caused by the directional flow of the precursor solution during the coaxial co-extrusion process.

The hydrogel fibers prepared in this experiment had high conductivity, which could provide a new idea for the preparation of PEDOT:PSS conductive polymer networks.

#### 2.2.2. Characterization of Anti-Freeze Hydrogel Fibers

Effect of Internal and External Phase Flow Rates on the Diameter of Hydrogel Fibers. During the preparation of core–shell hydrogel fibers by co-extrusion with coaxial needles, it was found that different flow rates of the inner and outer phases affected the diameter of the anti-freeze hydrogel fibers. 

**Figure 7:**
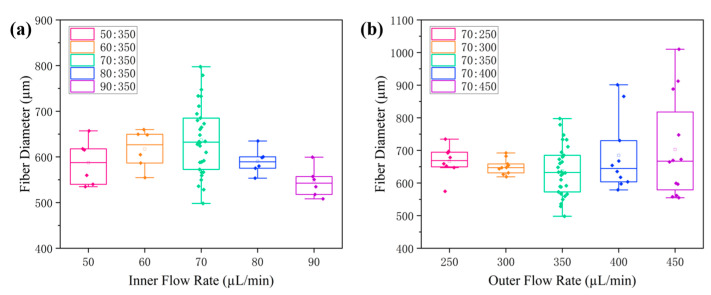
(**a**) Effect of internal phase flow rate on antifreeze hydrogel fiber diameter. (**b**) Effect of external phase flow rate on the diameter of antifreeze hydrogel fibers.

As shown in Figure 7a, when the outer-phase flow rate was maintained at 350 µL/min, the diameter of the core fibers was maximized with an inner-phase flow rate of 70 µL/min. As shown in Figure 7b, when the inner-phase flow rate was maintained at 70 µL/min, the diameter of the core fibers showed a minimum with an outer-phase flow rate of 350 µL/min.

Therefore, the diameter of the anti-freeze hydrogel fibers could be adjusted within a certain range by changing the flow rate of the inner and outer phases without changing the size of the coaxial needle. For the coaxial needle (1:5 cross-sectional area ratio), the inner- and outer-phase flow rates were 70 µL/min and 350 µL/min, respectively, for the subsequent experiments. The obtained hydrogel fibers had an average diameter of 604 µm, an electrical conductivity of 0.71 S/m, a tensile strength of 3.10 MPa, and an elongation at failure of 143%.

**Figure 8:**
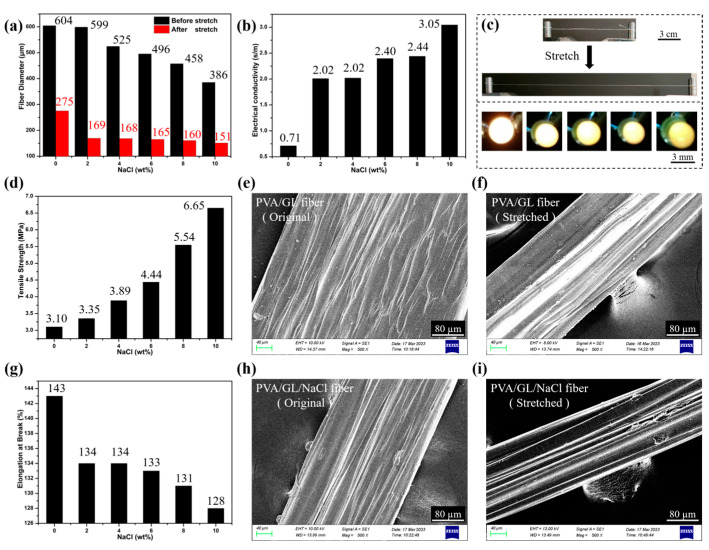
(**a**) Effect of NaCl content on the diameter of antifreeze hydrogel fibers. (**b**) Effect of NaCl content on the conductivity of antifreeze hydrogel fibers. (**c**) Effect of stretching process of antifreeze hydrogel fiber on the brightness of light-emitting diode. (**d**) Effect of NaCl content on the breaking tensile strength of antifreeze hydrogel fibers. (**e**) SEM observation of pristine PVA/GL antifreeze hydrogel fibers. (**f**) SEM observation of stretched PVA/GL antifreeze hydrogel fibers. (**g**) Effect of NaCl content on elongation at break of antifreeze hydrogel fibers. (**h**) SEM observation of pristine PVA/GL/NaCl antifreeze hydrogel fibers. (**i**) SEM observation of stretched PVA/GL/NaCl antifreeze hydrogel fibers.

Effect of NaCl Content on the Properties of the Hydrogel Fibers. As shown in Figure 8, with the increase in NaCl content, the hydrogel fibers were thinner (Figure 8a) and had higher electrical conductivities (Figure 8b) and tensile strengths (Figure 8d) but lower elongation strengths (Figure 8g). At 10 wt% NaCl, the fiber had a diameter of 386 µm, conductivity of 3.05 S/m, tensile strength of 6.65 MPa, and elongation at failure of 128%.

It had been shown that the addition of NaCl to PVA gels could produce a salting-out effect, which induced the intertwining of the PVA molecular chains and the formation of a more compact molecular network structure (Figure 8e,h), thus improving the mechanical properties of the gels, as well as increasing their electrical conductivity [42]. As shown in Figure 8c, the brightness of the light-emitting diode gradually decreased as the fibers were stretched, indicating that the resistance of the fibers gradually increased. After stretching, the anti-freeze hydrogel fibers had a denser structure and an oriented distribution (Figure 8f,i), so the anti-freeze hydrogel fibers could also be pre-stretched according to the application requirements.

The higher the NaCl content in the preparation process, the more viscous the gel solution was, which made the coaxial co-extrusion process difficult. In subsequent experiments, 2 wt% NaCl was chosen, resulting in an average fiber diameter of 599 µm, conductivity of 2.02 S/m, tensile strength of 3.35 MPa, and elongation at failure of 134%.

**Figure 9:**
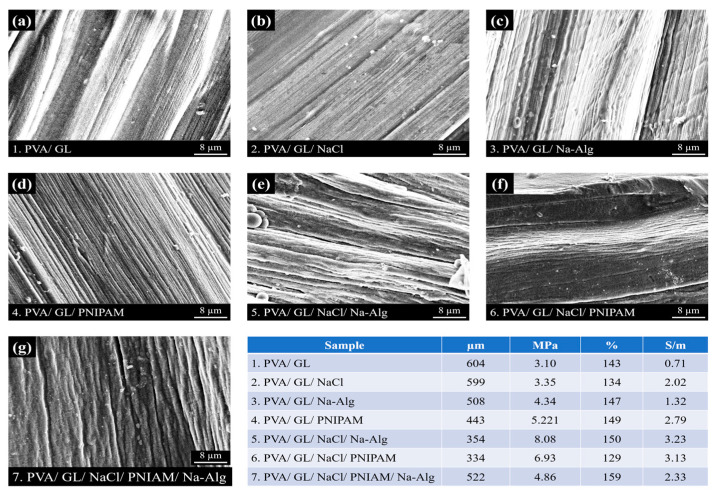
Diameter, electrical conductivity, and mechanical properties of fibers with different formulations. Microscopic morphology of PVA/GL fiber (**a**), PVA/GL/NaCl fiber (**b**), PVA/GL/Na-Alg fiber (**c**), PVA/GL/PNIPAM fiber (**d**), PVA/GL/NaCl/Na-Alg fiber (**e**), PVA/GL/NaCl/PNIPAM fiber (**f**), PVA/GL/NaCl/PNIPAM/Na-Alg fiber (**g**).

Effects of Different Functional Components on the Properties of Hydrogel Fibers. To improve the mechanical properties and electrical conductivity of the anti-freeze hydrogel fibers as well as provide environmental responsiveness, different functional components (NaCl, Na-Alg, and PNIPAM) were mixed in the inner-phase solution during the preparation process, and the microscopic morphology of anti-freeze hydrogel fibers of different formulations, as well as the optimal performance parameters, were shown in Figure 9.

In terms of electrical conductivity, SA and NaCl introduced a large number of conductive ions, and the electrical conductivity of PVA/GL/NaCl/Na-Alg fibers could reach up to 3.23 S/m. In terms of mechanical properties, the molecular chain segments of SA and PNIPAM were intertwined with the PVA gel network, which could enhance the tensile strength of the hydrogel fibers; in the synergistic effect of the salting-out effect, the tensile strength of PVA/GL/NaCl/Na-Alg fibers was up to 8.08 MPa, and the elongation at failure of PVA/GL/NaCl/PNIAM/Na-Alg fibers was up to 159%. PNIPAM could also endow the hydrogel fibers with temperature sensitivity. 

Therefore, the hydrogel fibers produced in this experiment had the functions of freeze resistance, moisturizing, conductivity, and temperature sensitivity, as well as the advantages of excellent mechanical properties, softness, transparency, and stretch-ability, which could be used in the fields of flexible sensing, bioelectronics, and transparent camouflage.

#### 2.2.3. Characterization of Hydrogel Fibers with Embedded Helical Channels

**Figure 10:**
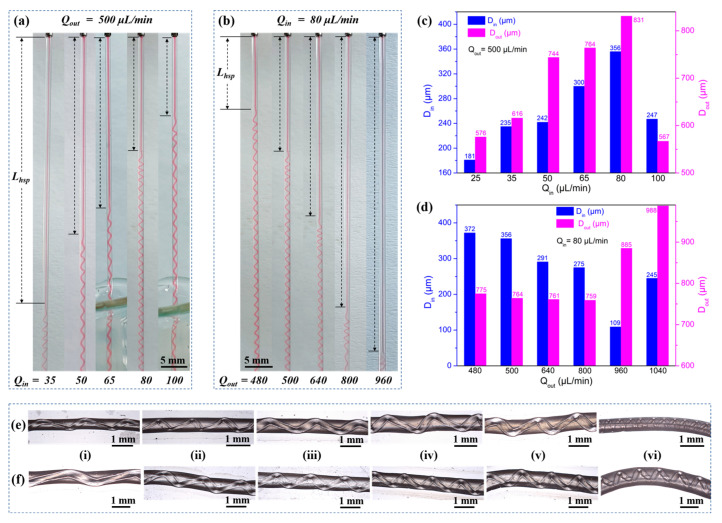
Effect of inner- and outer-phase flow rates on characteristic parameters: (**a**) The effect of increasing *Q_in_* on *L_hsp_* with fixed *Q_out_*. (**b**) The effect of increasing *Q_out_* on *L_hsp_* with fixed *Q_in_*. (**c**) The effect of fixed *Q_out_* and increasing *Q_in_* on *D_in_* and *D_out_*. (**d**) The effect of fixed *Q_in_* and increasing *Q_out_* on *D_in_* and *D_out_*. (**e**) The effects of fixed *Q_out_* and increasing *Q_in_* on *p*, where *Q_in_* for (**i**–**vi**) was 25, 35, 50, 65, 80, 100 μL/min in that order. (**f**) The effects of fixed *Q_in_* and increasing *Q_out_* on *p*, where *Q_out_* for (**i**–**vi**) was 480, 500, 640, 800, 960, and 1040 μL/min in that order.

Variation of Characteristic Parameters of Hydrogel Fibers with Embedded Helical Channels. As shown in Figure 10, the effects of inner- and outer-phase flow rates (Qout, Qin) on the helix generation position (*L_hsp_*), size (*D_out_*, *D_in_*), and pitch (*p*) were tested. *Q_ou_*_t_ was fixed at 500 µL/min, and *Q_in_* was gradually increased to test the changes on *L_hsp_* (Figure 10a), *D_out_* and *D_in_* (Figure 10c), and *p* (Figure 10e). Then, *Q_in_* was fixed at 80 µL/min, and *Q_out_* was gradually increased to measure the changes on *L_hsp_* (Figure 10b), *D_out_* and *D_in_* (Figure 10d), and *p* (Figure 10f). Within the ranges of *Q_in_* (25–100 µL/min) and *Q_out_* (480–1040 µL/min) tested, the following basic patterns were obtained.

1. *L_hsp_* decreased with a higher *Q_in_* but increased with a higher *Q_out_* (Figure 10a,b). The important factor to consider was the fluid interface where the high-viscosity Ca-Alg layer formed. For a constant Qout, increasing *Q_in_* led to faster intermediate layer formation and a shorter *L_hsp_*. However, with constant *Q_in_*, the time required for the ionic crosslinking reaction was constant. As *Q_out_* increased, helix formation began farther away from the exit of the coaxial needle.
2. Both *D_in_* and *D_out_* increased as *Q_in_* increased, with the exception of *Q_in_* = 100 µL/min (Figure 10c). However, when *Q_out_* was varied with a constant *Q_in_*, *D_in_* and *D_out_* behaved differently (Figure 10d). As *Q_out_* was increased, *D_in_* remained relatively constant before increasing at *Q_out_* = 960 µL/min. *D_out_* generally decreased as *Q_out_* increased.
3. The pitches of the helical channels varied without a clear pattern. This might be due to the fact that in addition to *Q_out_* and *Q_in_*, weak factors like the crosslinking reaction conditions, gravity, the force generated by the inner wall of the capillary glass tube, and the axial resistance generated by entry into the solidification receiving bath all had an influence on *p*. The current experimental conditions did not allow us to conduct control variable comparison tests for these weak factors.

**Figure 11:**
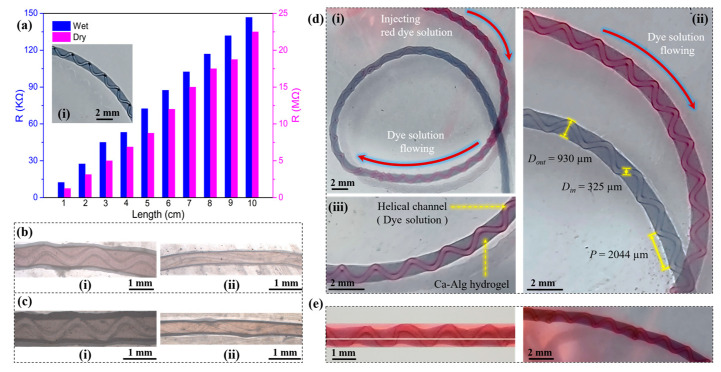
Conductivity, tensile, and circulation tests of hydrogel fibers with embedded helical channels: (**a**) The variation of resistance with length for hydrogel fibers in wet and dry states, where the inset figure (**i**) showed the physical appearance of hydrogel fibers with PEDOT:PSS added. (**b**) Local enlargements of hydrogel fibers before (**i**) and after stretching (**ii**). (**c**) Local enlargements of hydrogel fibers with PVA added before (**i**) and after (**ii**) stretching. (**d**) Long-distance injection flow performance of embedded helical channel hydrogel fibers (**i**), where (**ii**,**iii**) were local enlargements. (**e**) Slow release phenomenon of embedded helical channel hydrogel fibers.

Conductivity, tensile, and circulation tests of hydrogel fibers with embedded helical channels. As shown in Figure 11, the electrical conductivity, mechanical properties, and circulation properties of the embedded helical channel hydrogel fibers were tested. When aqueous PEDOT:PSS (1.1 wt%) was mixed with 2 wt% aqueous Na-Alg homogeneously as the outer phase, conductive helical channeled hydrogel fibers were produced. The conductive fibers showed an overall light blue color, while the embedded helical channels remained hollow and transparent (Figure 11a(i)). In both wet and dry states, the resistance increased linearly with an increase in length (Figure 11a), indicating that the fibers had stable structure and uniform conductivity. 

By adding 10 wt% of aqueous PVA to the outer-phase solution during the fiber preparation, the mechanical properties were improved for the produced conductive helical channeled hydrogel fibers. The addition of PVA to the outer phase increased the viscosity, which in turn caused an increase in *D_in_*. The measured and calculated *D_in_*, tensile strength, and elongation at failure of the hydrogel fibers before and after the addition of PVA were 220 µm, 0.479 MPa, 43.3%, and 296 µm, 0.495 MPa, 45.4%, respectively. The hydrogel fibers retained their embedded helical channel structure after stretching (Figure 11b,c).

The embedded helical channels were tested for red dye delivery ability. The flow of the red dye in the hydrogel fibers could be clearly observed through the helix (Figure 11d(i,ii)). Moreover, the red dye did not diffuse during the injection process, as the outer layer remained light blue (Figure 11d(iii)), indicating that the fabricated helical channeled hydrogel fibers had the potential to deliver fluid along a long distance, measured in decimeters and meters. The above hydrogel fibers were left to stand for 12 h. It was found that the red dye slowly diffused into the outer wall and the surrounding environment (Figure 11e). The fluid-transport and slow-release properties of the embedded helical channel hydrogel fibers showed potential for biomedical applications.

### 2.3. Application Summaries of Hydrogel Fibers in Sensing for Healthcare

As a typical temperature-sensitive polymer material, PNIPAM has a phase transition temperature (PTT) of about 32 °C, [53] which is around the human body temperature. For the linear hydrogel fibers with temperature sensitivity and electrical conductivity, the surface porous structure (Figure 6e) and the large aspect ratio gave it a large specific surface area. This could allow for the detection of changes in ambient temperature with applications in human respiratory monitoring. The anti-freeze hydrogel fibers prepared by double-layer hydrogel fibers had excellent moisture retention properties and could be used for higher temperature monitoring after being modified with PEDOT:PSS and PNIPAM.

#### 2.3.1. Thermosensitive Electrically Conductive Linear Hydrogel Fibers Used for Human Respiratory Monitoring

The hydrogel fibers were first left in the open for 48 h as the equilibrium state. Relative resistance changes were then measured after breathing on them. The breathing test was divided into two scenarios: normal breathing and wave breathing.

**Figure 12:**
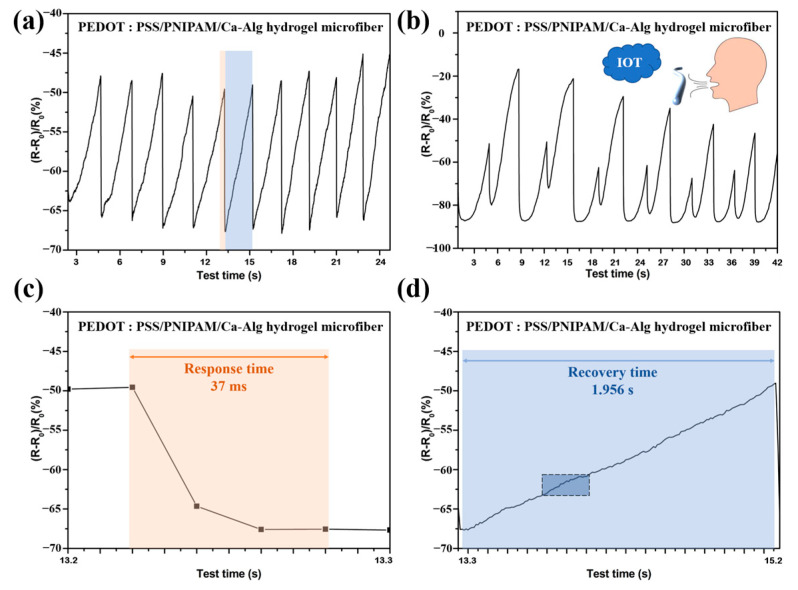
Thermosensitive and electrically conductive hydrogel fibers for human respiratory monitoring. Relative change rates of resistance of hydrogel fibers during (**a**) normal and (**b**) fluctuating breathing. (**c**) Response time and (**d**) recovery time for monitoring the change in hydrogel fiber resistance during normal breathing.

For the normal breathing scenario, when volunteers breathed on the hydrogel fibers, the resistance dropped sharply from the temperature of exhalation and then recovered rapidly (Figure 12a). As shown in Figure 12c–d, the respiration response time of PEDOT:PSS/ PNIPAM/Ca-Alg hydrogel microfibers was 37 ms while it took a longer time of 1.956 s to be recovered due to the relatively slow heat dissipation process in the room condition. This process showed that the hydrogel fibers respond very quickly to temperature changes, and the 10-cycles test proved that the reversibility and cyclability were good.

For the wave breathing scenario, the large aspect ratio of hydrogel fibers and the micro-porous structure of the surface (Figure 6e) allowed the hydrogel fibers to clearly distinguish the strength of breathing through the heights of the peaks (Figure 12b). Combined with Internet of Things (IOT) technology, it could be used for intelligent monitoring of human breathing and early warning of Sleep Apnea Syndrome (SAS).

#### 2.3.2. PVA/GL/PNIPAM Frost-Resistant Hydrogel Fibers as Temperature Sensors

It had been shown that the temperature sensitivity of PVA/PNIPAM fibers was provided by PNIPAM, and its “temperature-resistance” trend was related to the PNIPAM phase transition temperature (PTT, 32 °C) [53].

**Figure 13:**
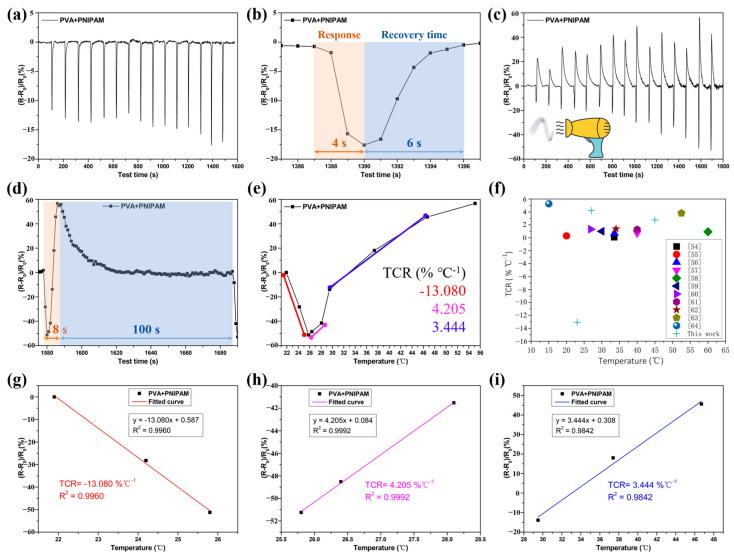
Anti-freeze hydrogel fibers for temperature sensing. Cycling tests for warming from 22 °C to (**a**,**b**) 30 °C and (**c**,**d**) 55 °C, and the respective response and recovery times. (**e**,**f**) TCR of hydrogel fibers and comparison with related research. (**g**–**i**) Fitted curves of TCR for different temperature intervals.

Below the PTT, PVA/PNIPAM resistance decreased when the ambient temperature increased from 21.9 °C to 29.5 °C (Figure 13a), with the resistance returning to baseline values as the temperature was cycled back to the starting temperature. This showed that the PVA/PNIPAM anti-freeze hydrogel fibers responded very quickly to temperature changes, and the 15-cycles test proved good reversibility and cyclability.

Above the PTT, the resistance rose rapidly as the temperature rose. As shown in Figure 13c, the ambient temperature increased from 21.9 °C to 55.1 °C; the PVA/PNIPAM resistance first decreased (as observed from the below PTT scenario) and then rapidly increased as the temperature exceeded the PTT. As the temperature returned to baseline values, the resistance returned to its starting point. The cyclic test was performed 15 times. It showed that rapid, reversible, and reusable temperature monitoring could be achieved by adding temperature-sensitive functional components to the anti-freeze hydrogel fibers.

As shown in Figure 13b,d, the response times of PVA/PNIPAM anti-freeze hydrogel fibers for low temperature (21.9 °C) and high temperature (55.1 °C) were 4 s and 8 s, respectively, and the recovery times were 6 s and 100 s, respectively. The response time was related to the warming process, and the recovery time was related to the heat dissipation process. The peak fluctuation of the relative resistance change in the cyclic test was related to the peak temperature fluctuation cycle.

The temperature response of PVA/PNIPAM anti-freeze hydrogel fibers was evaluated by the temperature coefficient of resistance (TCR), *TCR* = [(*R*-*R*_0_)/*R*_0_]/Δ*T*, where *R* is the instantaneous resistance at the test temperature, *R*_0_ is the initial resistance, and Δ*T* is the temperature difference. The greater the TCR value of the sample, the higher its sensitivity to temperature response. As shown in Figure 13e, the PVA/PNIPAM anti-freeze hydrogel fibers maintain the negative thermosensitive properties of PNIPAM. That was, its TCR value gradually decreased with increasing temperature, and its TCR was −13.080% °C^−1^ (21.9~25.8 °C, R^2^ = 0.996), 4.205%°C^−1^ (26.4~29.5 °C, R^2^ = 0.999), and 3.444% °C^−1^ (37.4~55.1 °C, R^2^ = 0.984), respectively. The fitted curves were shown in Figure 13g–i. As shown in Figure 13f, the TCR values of PVA/PNIPAM anti-freeze hydrogel fibers were significantly better than those of the relevant studies available so far [54,55,56,57,58,59,60,61,62,63,64].

## 3. Conclusions

Conventional coaxial co-extrusion devices are effective in preparing axially homogeneous hydrogel fibers, but it is still a great challenge to produce complex geometries. To tackle this problem, this study combined the microfluidic coaxial co-extrusion technology with simple and rapid chemical reactions and put forward an improved scheme. In this scheme, the calcium alginate hydrogel formed by fast ion crosslinking was taken as the hydrogel basis. Hydrogel fibers with complex structures and multiple functions were then generated by adjusting the compositions of the inner- and outer-phase solutions. The complex structures included single-layer linear fibers, core–shell structure fibers, fibers with embedded helical channels, hollow tube fibers, and necklace fibers. The multiple functions were electrical conductivity, temperature response, mechanical enhancement, anti-freeze, moisture retention, and liquidity. Due to the instantaneous ionic crosslinking reaction of Na-Alg with Ca^2+^ and the automatic winding device, the developed scheme could realize large-scale and automatic continuous production of multifunctional bionic hydrogel fibers.

The continuous preparation of PEDOT:PSS/PNIPAM/Ca-Alg thermosensitive and electrically conductive single-layered linear hydrogel fibers with IPN structure was achieved by using an inner phase of Na-Alg solution, PNIPAM hydrogel precursor solution, PEDOT:PSS, and aqueous CaCl_2_ as the outer phase and the receiving solidification bath. The conductivity of the gel fibers was recorded as up to 22.71 S/m. Additionally, a fast and reversible temperature response, useful for human respiration monitoring, was observed with a response time of 37 ms and a recovery time of 1.956 s. It could also detect between strong and weak pulses.

The core–shell hydrogel fibers with an inner-phase solution as the core and ionically crosslinked Ca-Alg as the shell was continuously prepared by using an anti-freeze hydrogel precursor solution as the inner-phase solution, Na-Alg as the outer-phase solution, and aqueous CaCl_2_ as the receiving coagulation bath. After co-extrusion, the core–shell hydrogel fibers were frozen, and the brittle Ca-Alg hydrogel shell was peeled off and removed to obtain the anti-freeze hydrogel fibers. By adjusting the inner- and outer-phase flow rates and the formulation of the inner-phase solution, the regulation of the diameter, electrical conductivity, mechanical properties, and temperature-responsiveness of the anti-freeze hydrogel fibers was achieved. The measured conductivity reached up to 3.23 S/m, tensile strength reached up to 8.08 MPa, elongation at failure reached up to 159%, and TCR reached up to −13.080% °C^−1^ (21.9~25.8 °C, R^2^ = 0.996). The gel fibers also had the advantages of being soft, transparent, stretchable, and biocompatible and could be applied to flexible sensing, bioelectronics, soft robotics, and transparent camouflage.

Being different from the above two hydrogel fiber preparations, the non-viscous CaCl_2_ solution as the inner phase and the viscous Na-Alg solution as the outer phase, combined with the heterogenerated coiled rope effect, allowed for the continuous preparation of Ca-Alg hydrogel fibers with embedded helical channels. The characteristic parameters of the hydrogel fibers (*L_hsp_*, *D_in_*, and *D_out_*) could be adjusted by the *Q_in_* and *Q_out_*. *L_hsp_* decreased with a higher *Q_in_* but increased with a higher *Q_out_*. *D_in_* increased and then decreased as the *Q_in_* increased, and decreased and then increased as the *Q_out_* increased. *D_out_* followed a similar trend to that of *D_in_*. Furthermore, the above process was modified by using water as the inner-phase solution to create hollow tube hydrogel fibers or oil as the inner-phase solution for necklace-shaped hydrogel fibers. The hydrogel fibers with hollow structure can be used in biomimetic applications such as artificial blood vessels, controlled delivery systems, flexible drive systems, and biological scaffolds.

## 4. Materials and Methods

### 4.1. Materials

Poly (3,4-ethylenedioxythiophene): poly (4-styrenesulfonate) (PEDOT:PSS, 1.1 wt%, 700 S/cm) was purchased from Shanghai O-Yi Organic Optoelectronic Materials Co., Ltd. (Shanghai, China). N-Isopropylacrylamide monomer (NIPAM, purity ≥ 98.0%, with stabilizer MEHQ), N,N′-methylene bisacrylamide (BIS, AR), potassium persulfate (KPS, GR, purity ≥ 99.9%), N′N′N′N′-tetramethylethylenediamine (TEMED, purity ≥ 99.5%), sodium alginate (Na-Alg, purity ≥ 99.5%), calcium chloride (CaCl_2_, purity ≥ 96%), sodium chloride (NaCl, AR, purity ≥ 99.5%), polyvinyl alcohol (PVA, alcoholysis 87.0~89.0% mol/mol) and glycerol (GL, AR, purity ≥ 99%) were purchased from Shanghai Maclin Biochemistry Technology Co., Ltd. (Shanghai, China). All chemical reagents were used as received without further purification.

### 4.2. Preparation Method of Multifunctional Biomimetic Hydrogel Fibers

**Figure 14:**
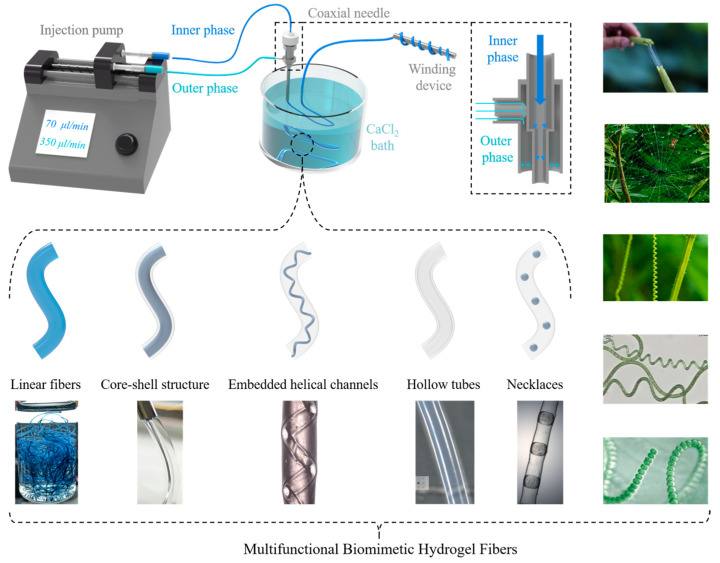
Schematic diagram showing the preparation of multifunctional biomimetic hydrogel fibers.

This study designed a microfluidic coaxial co-extrusion device, as shown in Figure 14. By adjusting the compositions and flow rates of the internal and external phase solutions, linear hydrogel fibers (temperature responsive and electrically conductive), core–shell structure hydrogel fibers (anti-freeze and moisturizing), and hydrogel fibers with embedded helical channels, hollow tubulars, and microspheres were prepared. 

Sodium alginate (Na-Alg) aqueous solution was used, with PNIPAM gel precursor solution, PEDOT:PSS as the inner phase, and calcium chloride (CaCl_2_) aqueous solution as the outer phase. A Ca-Alg ionically crosslinked network was first formed by the rapid reaction between Na-Alg and Ca^2+^ to ensure the preliminary formation of hydrogel fibers, which could allow the continuous preparation of linear hydrogel fibers. Then, a PNIPAM covalently crosslinked network was formed by free radical polymerization inside the initially formed hydrogel core–shell fibers, so that PEDOT:PSS/PNIPAM/Ca-Alg conductive temperature-sensitive hydrogel fibers with an interpenetrating polymer network (IPN) could be produced. 

After verifying the feasibility of the rapid preformation of “microfluidic control combined with ionic crosslinking” for the continuous preparation of hydrogel fibers, the formulation of the precursor solution and the preparation process were adjusted to increase freeze resistance. The freeze resistance hydrogel precursor solution utilized a water/glycerol mixture as the inner-phase solution, Na-Alg as the outer-phase solution, and CaCl_2_ as the receiving solidification bath. Core–shell hydrogel fibers with an inner-phase solution as the core and ionically crosslinked Ca-Alg as the shell were formed by coaxial co-extrusion. PVA/GL anti-freeze hydrogel fibers were obtained by freezing the core–shell hydrogel fibers and peeling off the frozen shell. 

Finally, Ca-Alg hydrogel fibers with embedded helical channels were successfully prepared by using a calcium chloride inner-phase solution in combination with microfluidic technology and heterogeneous rope coil effects. The use of water as the inner-phase solution produced empty tubular hydrogel fibers, and the use of oil as the inner-phase solution produced necklace-shaped hydrogel fibers.

## Data Availability

The original contributions presented in the study are included in the article/Supplementary Material. Further inquiries can be directed to the corresponding author/s.

## Supplementary Materials

The following supporting information can be downloaded at: https://www.mdpi.com/article/10.3390/gels10090590/s1, Figure S1: Photos of preparation process of the core–shell structure hydrogel fibers; Figure S2: Photos of the core–shell hydrogel fibers before and after freezing; Figure S3: Photos of preparation process of the hydrogel fibers with embedded helical channels; Figure S4: Photos of PNIPAM/Ca-Alg hydrogel microfibers and PEDOT:PSS/PNIPAM/Ca-Alg hydrogel microfibers (PDF); Movie S1: Continuous preparation of the core–shell hydrogel fibers (MP4); Movie S2: Automatic coiling process of the core–shell hydrogel fibers (MP4); Movie S3: Continuous preparation of the hydrogel fibers with embedded helical channels (1) (MP4); Movie S4: Continuous preparation of the hydrogel fibers with embedded helical channels (2) (MP4).
