# Supplementary material for: Facile Formation of Multifunctional Biomimetic Hydrogel Fibers for Sensing Applications
Source: Gels. 2024 Sep 13;10(9):590. doi: 10.3390/gels10090590 (PMC11431008; doi:10.3390/gels10090590)
Supplement: Supplementary file 1 [file gels-10-00590-s001.zip › Supporting Information.pdf]

Supporting Information

Facile Formation of Multifunctional Biomimetic Hydrogel Fibers for Sensing Applications

Mengwei Jia [a], *Mingle Guan* [a], *Ryan Yao* [b], *Yuan Qing* [a], *Xiaoya Hou* [a, c], *Jie Zhang* [a, c, d] *

[a] School of Mechanical Engineering, Jiangnan University, No.1800, Lihu Avenue, Wuxi City, Jiangsu 214122, P.R. China

[b] College of Engineering, University of Illinois at Urbana-Champaign, Illinois USA

[c] Jiangsu Key Laboratory of Advanced Food Manufacturing Equipment and Technology (Jiangnan University)

[d] Advanced Technology Center, Wuxi, China

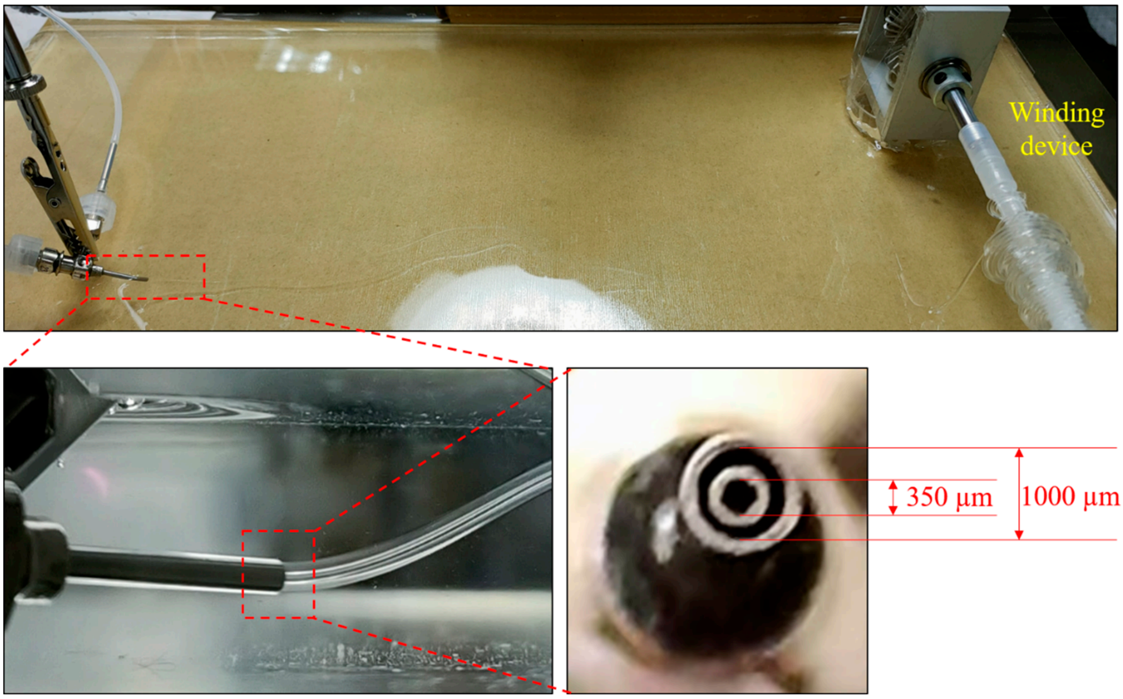

Figure S1. Photos of preparation process of the core-shell structure hydrogel fibers.

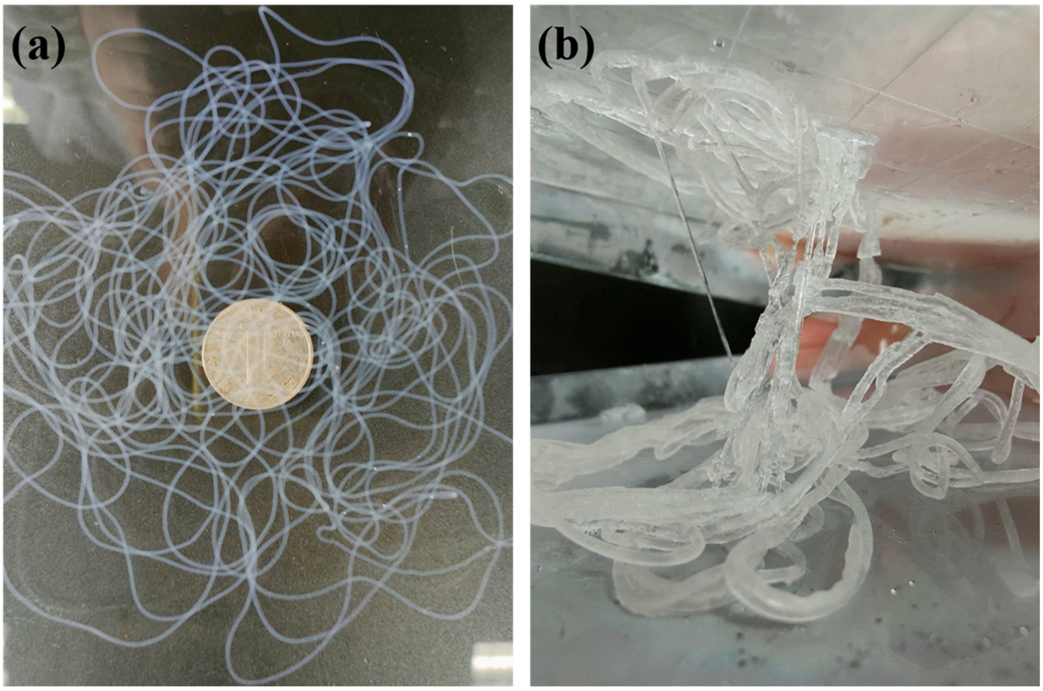

Figure S2. Photos of the core-shell hydrogel fibers before (a) and after (b) freezing.

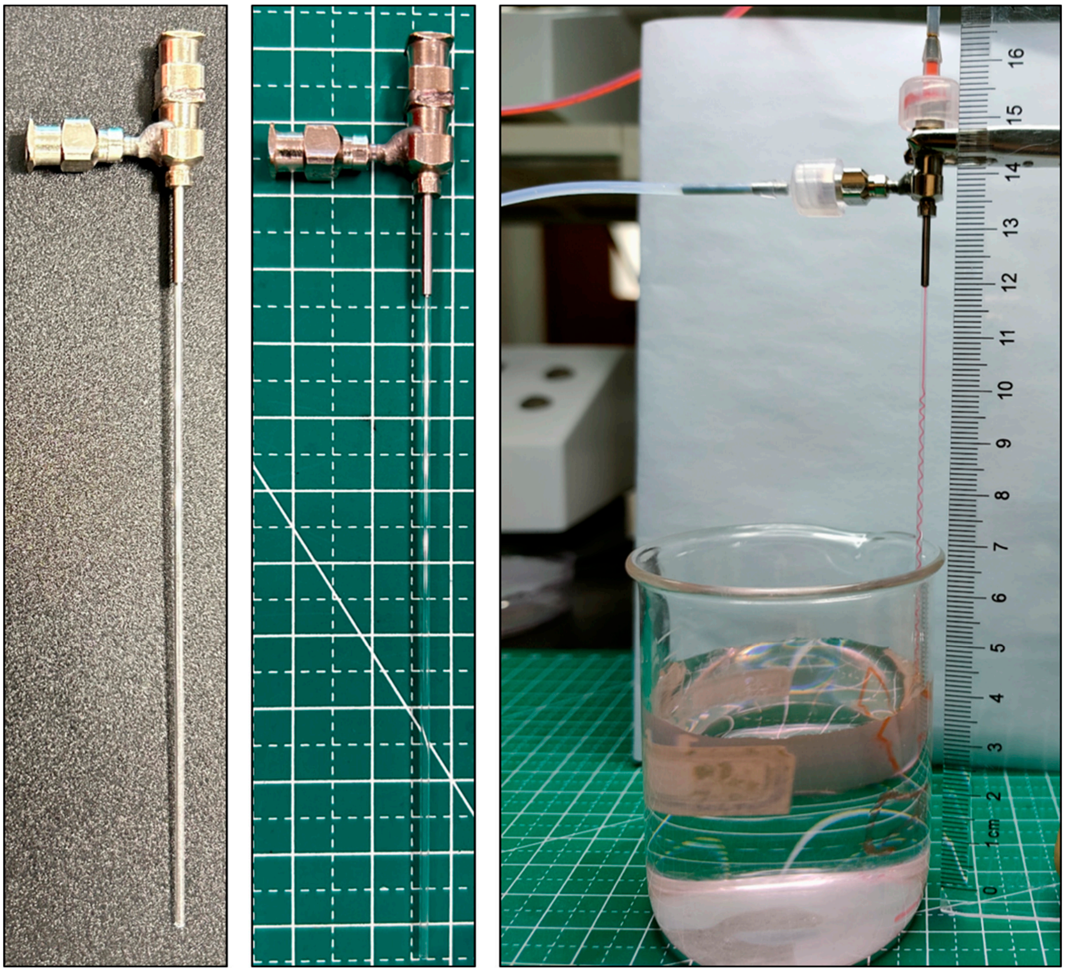

Figure S3. Photos of preparation process of the hydrogel fibers with embedded helical channels.

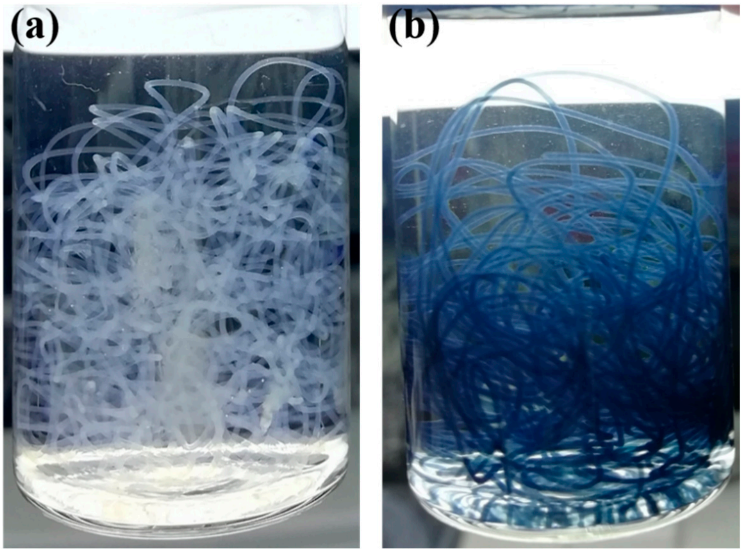

Figure S4. Photos of PNIPAM/Ca-Alg hydrogel microfibers (a) and PEDOT: PSS/PNIPAM/Ca-Alg hydrogel microfibers (b).

Movie S1. Continuous preparation of the core-shell hydrogel fibers.

Movie S2. Automatic coiling process of the core-shell hydrogel fibers.

Movie S3. Continuous preparation of the hydrogel fibers with embedded helical channels (1).

Movie S4. Continuous preparation of the hydrogel fibers with embedded helical channels (2).
